# Design, Synthesis, Antimicrobial Evaluation and Molecular Modeling Study of 1,2,4-Triazole-Based 4-Thiazolidinones

**DOI:** 10.3390/molecules21050568

**Published:** 2016-04-30

**Authors:** Sahar Ahmed, Mohamed F. Zayed, Shahenda M. El-Messery, Mohamed H. Al-Agamy, Hamdy M. Abdel-Rahman

**Affiliations:** 1 Department of Pharmacognosy and Pharmaceutical Chemistry, College of Pharmacy, Taibah University, Al-Madinah, Al-Munawarah 30001, Saudi Arabia; mfzayed25@yahoo.com (M.F.Z.); shahenda76@yahoo.com (S.M.E.-M.); 2 Department of Medicinal Chemistry, Faculty of Pharmacy, Assiut University, Assiut 71526, Egypt; hamdym01@yahoo.com; 3 Pharmaceutical Chemistry, Department of Pharmaceutical Chemistry, Faculty of Pharmacy, Al-Azhar University, Cairo 11884, Egypt; 4 Department of Pharmaceutical Organic Chemistry, Faculty of Pharmacy, Mansoura University, Mansoura 35516, Egypt; 5 Department of Pharmaceutics, College of Pharmacy, King Saud University, P. O. Box 2457, Riyadh 11451, Saudi Arabia; elagamy71@yahoo.com; 6 Department of Microbiology and Immunology, Faculty of Pharmacy, Al-Azhar University, Nasr City, Cairo 11884, Egypt

**Keywords:** 4-thiazolidinones, 1,2,4-triazoles, antibacterial agents, antifungal agents, molecular docking

## Abstract

A series of 3-(2*H*-1,2,4-triazol-5-yl)-1,3-thiazolidin-4-one derivatives (**7c**–**l**) was designed and synthesized. Their structures have been elucidated based on analytical and spectral data. They were evaluated for their antibacterial and antifungal activities. Compound **7h** showed the highest activity against all tested strains, except *P. vulgaris*, with MIC 8 μg/mL and 4 μg/mL against *S. aureus* and *C. albicans*, respectively. Furthermore, Compounds **7c**, **7h**, and **7j** demonstrated moderate anti-mycobacterium activity. The binding mode of the synthesized thiazolidinones to bacterial MurB enzyme was also studied. Good interactions between the docked compounds to the MurB active site were observed primarily with Asn83, Arg310, Arg188 and Ser82 amino acid residues.

## 1. Introduction

Despite the significant advances in the field of antimicrobial agents ever since the discovery of penicillin, infections are still the second-leading cause of death worldwide and remain an important public health problem [1,2]. Consequently, the rapid emergence of drug-resistant strains of different bacterial pathogens, particularly *Mycobacterium tuberculosis* and *Staphylococcus aureus*, highlights the need for new classes of antimicrobial agents [3]. These new classes would either be of a different chemical structure or a modified structure compared to those of existing agents [4]. Peptidoglycan is an essential component of the bacterial cell wall. It confers mechanical resistance to higher internal osmotic pressure and maintains a defined cell shape [5]. Therefore, drugs interfering with the biosynthesis and assembly of peptidoglycan are effective antimicrobial agents. The structure of peptidoglycan is unique to prokaryotic cells and, thus, optimal for the selective targeting of vital microbial pathways [6].

**Figure 1:**
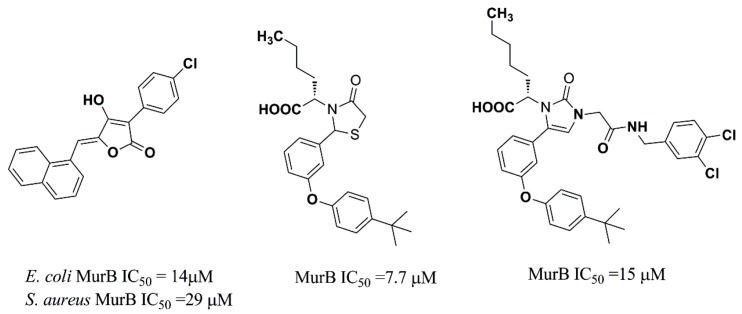
Examples of small molecule MurB inhibitors with their reported antibacterial activity.

Uridine diphosphate UDP-*N*-Acetylenolpyruvylglucosamine reductase (Mur) enzymes are integral components in bacterial peptidoglycan biosynthesis that provide valuable targets for specific bacterial cell wall inhibitors. There is a growing interest in MurB inhibitors, as it is essential for the viability of bacterial cells without any homologue in eukaryotic cells. A variety of MurB small molecule inhibitors with antibacterial activity have already been reported (Figure 1) [7]. Substituted 4-thiazolidinones and their analogues working as MurB inhibitors have received great attention. Trisubstituted 4-thiazolidinones inhibited the *Escherichia coli* MurB enzyme at a low micromolar level, most probably acting as diphosphate surrogates for the natural substrate (Figure 1) [8]. The related imidazolidinone also inhibited MurB enzyme, showing good antibacterial activity (Figure 1) [9].

**Figure 2:**
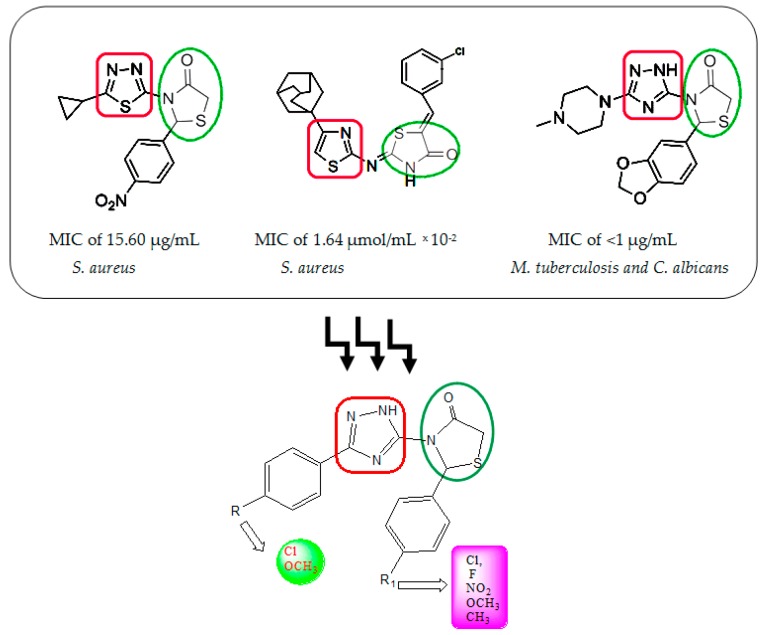
Rationale and design of title Compounds **7c**–**l** based on the thiazolidin-4-one and the triazole scaffold.

The SAR of the antibacterial activity of the 1,3-thiazolidin-4-one scaffold has become the subject of intense research projects [10,11]. Exploration of the 1,3-thiazolidin-4-ones showed that their antibacterial activity not only depended on the 1,3-thiazolidin-4-one pharmacophore, but also on the nature and position of its substituents; for example, electron withdrawing group substitutions on aromatic rings of biphenyl-4-thiazolidinone derivatives showed improved antibacterial activity [11]. Numerous new derivatives with the pharmacophoric 4-thiazolidinone ring were substituted with five-membered heterocyclic rings, [12] such as thiadiazole, [13] thiazoles [14] or 1,2,4-triazoles [15], as seen in Figure 2. Moreover, 1,2,4-triazoles are core structures in medicinal chemistry research with diverse biological activities [16,17,18].

Based on the studies mentioned above, and in continuance of our previous work [15], herein, we describe the rational design, synthesis and characterization of new substituted 4-thiazolidinone-1,2,4-triazole hybrids towards searching for new compounds of promising antimicrobial activity. The synthesized compounds were evaluated against different Gram-positive and Gram-negative bacteria strains. Their antimycobacterial activity was evaluated against *M. fortuitum*. The prepared compounds were also evaluated for their antifungal activity against *Candida albicans*. Additionally, molecular modeling studies were also carried out to shed light on the binding mode of the synthesized compounds to the MurB enzyme by docking into the crystal structure of *S. aureus* MurB (PDB 1HSK) [19].

## 2. Results and Discussion

### 2.1. Chemistry

**Scheme 1:**
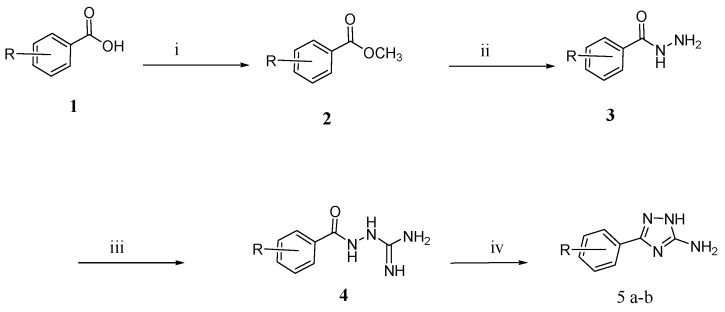
Synthesis of starting compounds (**5a**,**b**). *Reagents and conditions*: (**i**) HCl gas, methanol; (**ii**) H_2_NNH_2_, absolute ethanol, reflux 5 h; (**iii**) S-methylisothiourea sulfate; (**iv**) 5% aqueous NaOH reflux 3 h; neutralize with HCl.

**Scheme 2:**
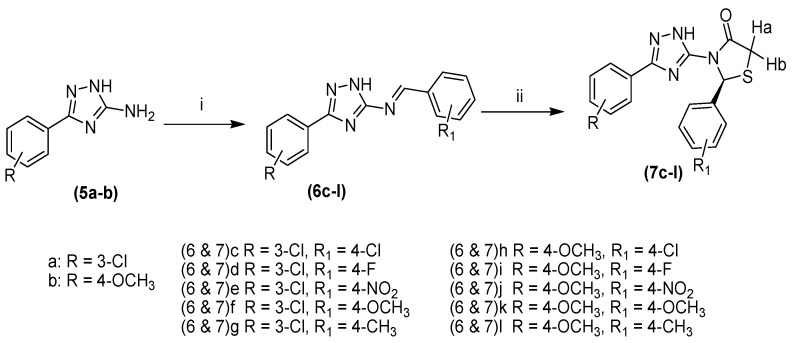
Synthesis of target compounds (**7c**–**l**); *Reagents and conditions*: (**i**) Ar-CHO, HOAc, reflux 10–12 h; (**ii**) mercaptoacetic acid, THF, pinch ZnCl_2_; reflux, 12 h.

Simple and straightforward synthetic procedures were adopted for the synthesis of our target 4-thiazolidinone derivatives. The key intermediates 3-amino-5-substituted-1,2,4-triazoles **5a**–**b** were synthesized in accordance with the previously-developed chemistry (Scheme 1) [20,21]. Synthesis of 1,3-thiazolidin-4-ones involves three main components: an amine, a carbonyl compound and a mercapto-acid, the synthetic process either prepared in a one-pot three-component condensation [15] or in a two-step process, which we followed (Scheme 2). The reaction pathway involves the *in situ* formation of intermediate 5-triazolylimines (**6c**–**l**) by condensation of the amine and the appropriate aldehydes [22]. The produced imines **6c**–**l** undergo attack by generated sulfur nucleophile when refluxed with mercaptoacetic acid followed by the intramolecular cyclization to yield the substituted 1,3-thiazolidin-4-ones **7c**–**l**. All of the synthesized compounds were characterized by spectroscopic techniques (^1^H-NMR, ^13^C-NMR and mass spectra), as well as elemental analyses. The spectral data of the synthesized compounds (**7c**–**l**) were in agreement with their proposed structures. The IR data clearly showed a strong C=O stretching band around 1730 cm^−1^ and 1259 cm^−1^, which were characteristic for 4-thiazolidinones, in addition to the aromatic C-H absorption band around 3030 cm^−1^ along with the absence of the characteristic fork-shaped band of the free amino group for **5a**–**b**. The ^1^H-NMR data indicated the presence of characteristic peaks for 4-thiazolidinone protons of the doublet peak CHb of CH_2_ around 4.15 ppm and a doublet of the doublet peak for CHa at around 4.08 ppm of CH_2_ for 4-thiazolidinone. A more downfield doublet peak of the CH proton of the 4-thiazolidinone ring was observed at about 5.6 ppm. Characteristic peaks for aromatic protons appear in their expected range of 6–7 ppm. ^13^C-NMR of synthesized compounds showed characteristic peaks for carbon 2 and carbon 5 of the thiazolidinone ring at their expected chemical shift δ 34 and 73 ppm, respectively, while its C4 appeared as a downfield signal at around δ 170 ppm.

### 2.2. Biological Studies

#### 2.2.1. Antimicrobial Activity

Primary screening for antimycobacterial activity of all newly-synthesized compounds was performed against *Mycobacterium fortuitum* at a concentrations ranging from 0.0625–128 μg/mL using moxifloxacin as the standard drug (Table 1). Compound **7h** was found to be the most active against *Mycobacterium fortuitum* with an MIC value of 32 μg/mL with a diameter of growth of inhibition zone (IZ) of 25 mm compared to 26 mm (IZ) of moxifloxacin. On the other hand, Compounds **7c**–**j** showed variable activities. Weak activity was observed for Compounds **7d**–**g** with an IZ range from 9–12 mm. Good activities were observed for Compounds **7c**, **7j** and **7h** with the IZ varying from 20–25 mm with an MIC between 32 and 64 μg/mL. Different variations on R and R1 gave compounds with a range of antimycobacterial activity. The antimycobacterial activity of the synthesized compounds against *Mycobacterium fortuitum* was observed when R was 4-methoxy, and R1 groups were in the order: 4-chloro > 4-nitro > 4-fluoro. Furthermore, when R was 3-chloro, R1 groups show in the order: 4-chloro > 4-methoy > 4-nitro and 4-fluoro (Scheme 2).

#### 2.2.2. Antibacterial Activity

**Figure 3:**
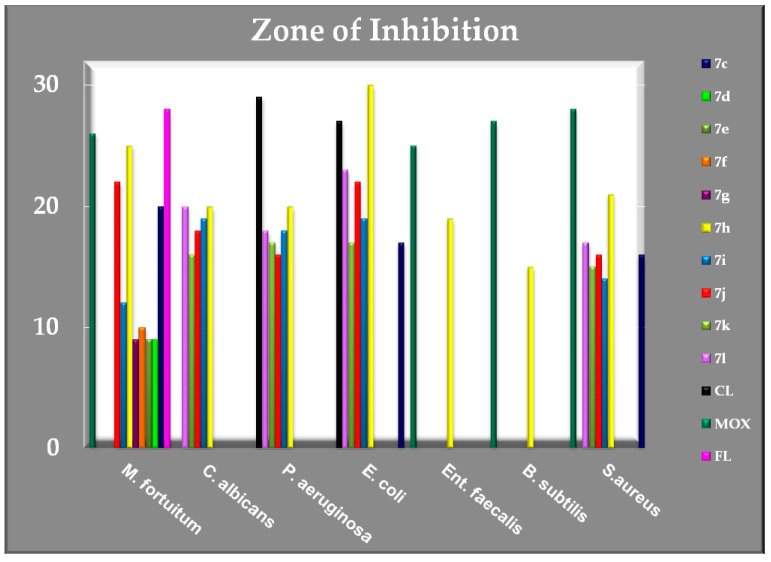
Graphical representation of the zone of inhibition (mm) of the compounds (**7c**–**l**) and reference antimicrobial drugs against different bacterial and fungal strains.

Antibacterial activities of Compounds **7c**–**l** were evaluated against six different groups of bacterial strains. The utilized microorganisms were *Enterococcus faecalis*, *Bacillus subtilis* and *Staphylococcus aureus* as Gram-positive strains and *Pseudomonas aeruginosa*, *Proteus vulgaris* and *Escherichia coli* as Gram-negative strains. Ciprofloxacin and Moxifloxacin were used as positive control drugs. The antibacterial activity was assessed using both the inhibition zone (IZ) and the minimal inhibitory zone (MIC). As shown in Table 1, Compounds **7h**–**l** showed moderate to very good broad-spectrum antibacterial activity, while Compounds **7d**–**g** showed no activity against all tested strains. Furthermore, it was noted that none of the tested compounds showed activity against the Gram-negative microorganism *Proteus vulgaris.* Compound **7h** showed the highest activity, and it was effective against all tested strains, except *Proteus vulgaris.* This compound was the most active against *Escherichia coli* with an inhibition zone (IZ = 30 mm) greater than the standard drug ciprofloxacin (IZ = 27 mm) at the same tested concentration. Compound **7c** showed moderate activity with ~61% inhibition zone compared to the standard drugs against *Staphylococcus aureus* and *Escherichia coli*. The MIC, as well as the zone of inhibition values for each compound are explained in Table 1 and Figure 3. All MIC values of the active compounds were found to be higher than those of ciprofloxacin and moxifloxacin. Most of the 1,2,4-triazole derivatives described here revealed activity that was found to have good MIC values (8–128 μg/mL), as shown in Table 1. The best MIC value observed for **7h** was 8 μg/mL against *Staphylococcus aureus*. Lipophilicity is an important physicochemical parameter in the development of an antibacterial agent, because it is known to be closely related to the permeation through the lipid coat of bacteria. The title compounds showed very good lipophilic characteristics with *p*-values of 3.4–5.2 (Table 1). From the structural point of view, it seems that the presence of a methoxy substituent at position R and fluorine, nitro, methoxy and methyl at the R1 position produce derivatives with promising antibacterial activity (Scheme 2). The 3-chloro substituent at position R produced promising antibacterial activity when R_1_ = Cl, while other substitutions did not give any antibacterial activity.

#### 2.2.3. Antifungal Activity

Preliminary screening of Compounds **7c**–**l** revealed good antifungal activity of the five derivatives **7h**–**l** against *Candida albicans*. The best activity was observed for Compounds **7h** and **7l** with IZ of 20 mm followed by **7i** (IZ = 19 mm) then **7g** (IZ = 18 mm), while **7k** showed the least activity with IZ of 16 mm compared to the 28-mm IZ of the standard drug fluconazole (Figure 3). The MIC of Compound **7h** against *Candida albicans* is 4 μg/mL compared to 0.5 µg/mL of fluconazole. On the other hand, Compounds **7i**–**l** showed MIC values of 16 μg/mL, whereas the rest of the compounds did not show any antifungal activity at the tested concentration (Table 1).

#### 2.2.4. Molecular Modeling Study

The X-ray crystallographic data of MurB, with UDP-sugar substrate, were used as guide surrogates of the diphosphate moiety. MurB confirmed that both substrates use the same binding pocket of the enzyme; this result is consistent with the role of the bound FAD cofactor in mediating hydride transfer from the NADPH to the UDP-sugar substrate [23]. The maximum number of poses per ligand was set to 30, and no constraints were used to perform molecular docking. The docking complex assumed to represent ligand-receptor interactions was selected based on three criteria: (i) binding affinity score of the pose; (ii) its orientation into the active site in a similar manner as the co-crystallized ligands orientation; and (iii) the preservation of binding interactions, especially hydrogen bond formation and lipophilic contacts. The estimated binding affinity of the co-crystallized ligand was −13.5795 Kcal/mol with of complex hydrogen network with a plethora of amino acids including, Arg310, Val199, Ala117, Gly153, Gly70, Asn83, Ser142, Ser82, Arg188, Gly133 and Arg228, giving a clue about the importance of hydrogen bond formation for effective enzyme binding.

**Figure 4:**
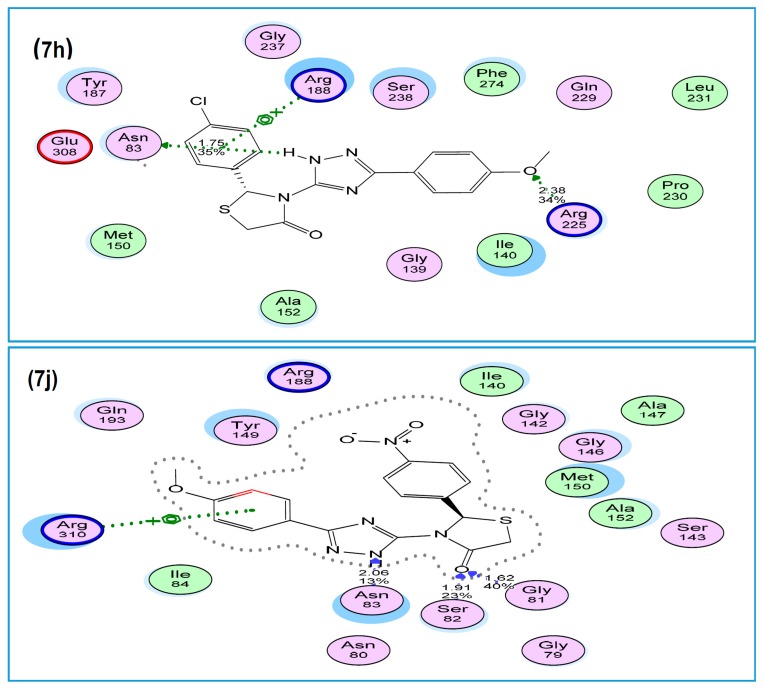
2D binding mode interaction and residues involved in the recognition of the most promising Compounds **7h** and **7j** into MurB binding site interaction showing the percentage of hydrogen bonds.

**Figure 5:**
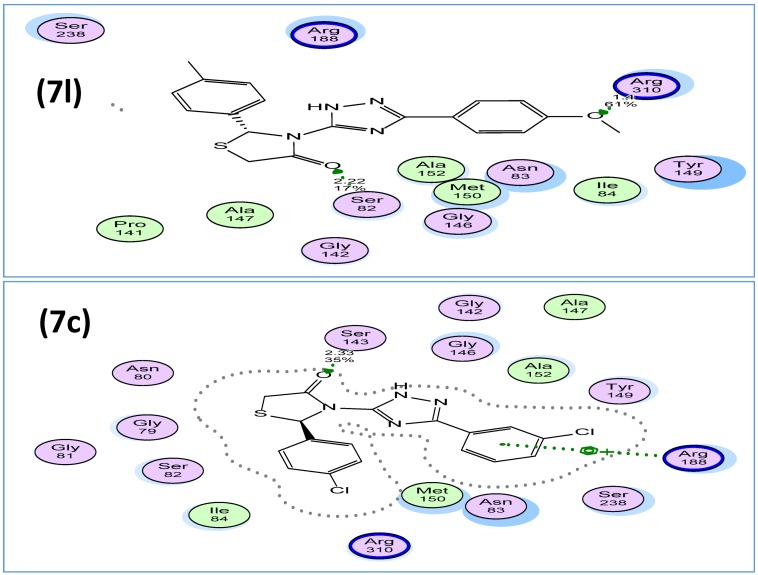
2D binding mode interaction and residues involved in the recognition of moderate active Compounds **7l** and **7c** into the MurB binding site interaction showing the percentage of hydrogen bonds.

**Figure 6:**
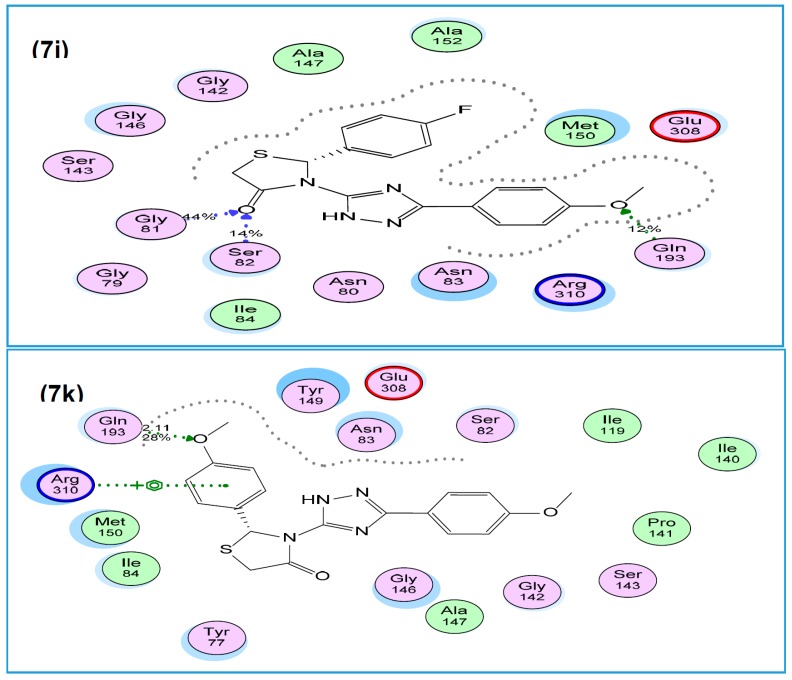
2D binding mode interaction and residues involved in the recognition of moderate active Compounds **7i** and **7k** into the MurB binding site interaction showing the percentage of hydrogen bonds.

The synthesized compounds were docked into the binding site of the MurB enzyme pocket and will be presented according to their better MIC values; however, there was a lack of accordance with matching MIC, which is logical, since the MIC is complex and unrelated to specific inhibition to any target enzyme. Modeling studies suggest that Compound **7h** binds to the MurB active site with a binding affinity of −12.18234 Kcal/mol via the hydrogen bonding interaction of the NH of triazole ring group with Asn83 with (36%, 1.75 Å) and Arg225 with the oxygen of methoxy group with (34% 2.38 Å), while it also shows cationic-arene interaction with the Arg188 residue (Figure 4). Compound **7j** showed a hydrogen bond interaction network with a triad of amino acids Asn83 (13%, 2.06 Å), Ser82 (23%, 1.91 Å) and Gly81 (40%, 1.61 Å) via the NH group of the triazole ring and the carbonyl group of the thiazolidine ring, respectively, with a binding affinity of −12.81985 Kcal/mol; in addition to a cationic arene interaction with the aromatic ring with the Arg310 amino acid (Figure 4). Compound **7l** has a binding affinity of −12.42222 Kcal/mol through the hydrogen bond interaction of (61%, 1.4 Å) and (17%, 2.22 Å) with Arg310 and Ser82 via methoxy oxygen and the carbonyl group of the thiazolidine ring, respectively (Figure 5). Compound **7c** gives two kinds of interactions: one is the hydrogen bonding interaction of (35%, 2.32 Å) with the Ser143 amino acid; the second is a cationic arene interaction with Arg188 (Figure 5) with a binding affinity of −11.64368, while Compound **7i** showed a triad hydrogen bonding interaction with Gly81, Ser82 and Gln193 with 44%, 14% and 12%, respectively (Figure 6).

**Figure 7:**
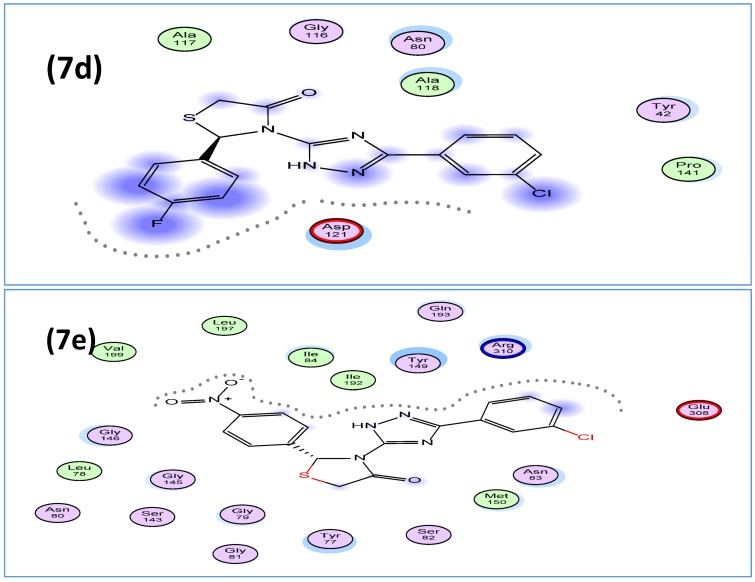
2D binding mode interaction and residues involved in the recognition of the non-active Compounds **7d** and **7e**, into the MurB binding site.

Compound **7k** provided a hydrogen bond interaction via Gln193 (28%) and Arg310 a cationic-arene interaction with a binding energy of −12.71658 Kcal/mol (Figure 6). On the other hand, Compounds **7d** and **7e** are examples of inactive antibacterial agents that did not show any binding to the MurB active site (Figure 7).

The docking study indicated that the important interactions of the compounds under study have resemblance to the binding mode with the FAD flavin ring system co-crystallized with enzyme with Asn83 and Arg310 or Arg188 and Ser82 amino acids, so they appear to provide a structural platform resulting in the tested compound’s proximity to FAD and its hydrogen bond to the main chain of these amino acids.

Analysis of the docking results suggests that the promising antimicrobial activity of the tested compounds could be attributed to: (i) higher binding affinity, but still less than the reference ligand; (ii) the incorporation with amino acids is similar to the co-crystallized ligand, especially the hydrogen bonding interactions. These findings are considered encouraging, providing a possible mechanism of action for the tested compounds and indicating the possibility of developing novel antibacterial agents targeting an early step in peptidoglycan biosynthesis.

**Figure 8:**
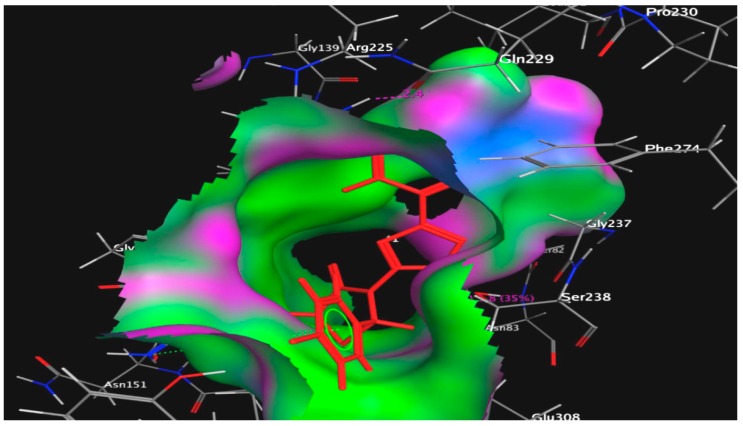
Surface map for the MurB active site with the docked conformation of Compound **7h** (red stick) (pink: hydrogen bond; blue: mild polar; green: hydrophobic region).

**Figure 9:**
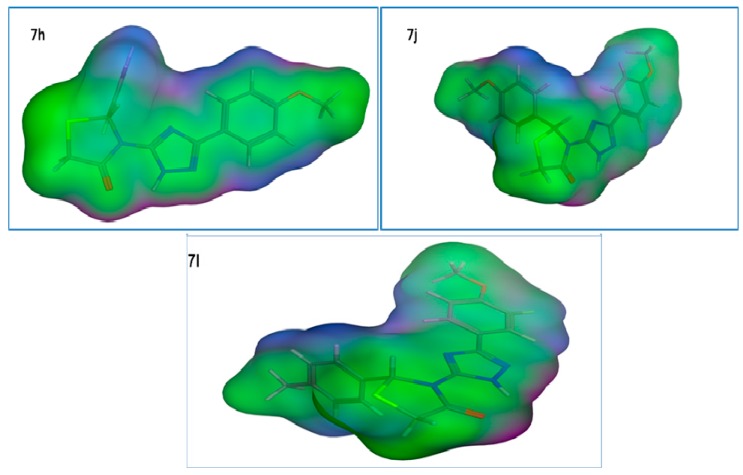
Surface map for Compounds **7h**, **7j** and **7l** (pink: hydrogen bond; blue: mild polar; green: hydrophobic region).

It was reported that the lipophilic characteristic of the previously-tested compounds could play an important role in their binding to the MurB active site [24]. Therefore, it was interesting to perform a deeper investigation of our compounds for the MurB binding site by studying the hydrophobic surface mapping of some tested candidates to get a clue about their lipophilic characteristic, which may imply their binding affinity to the binding pocket. In Figure 8, the surface map of the active site for the selected MurB enzyme along with the docked conformation of Compound **7h** are illustrated, showing a higher space of greener areas in the enzyme binding pocket, indicating a highly lipophilic active site. Figure 9 shows the surface mapping of some promising antimicrobials, Compounds **7h**, **7j** and **7l**, where the phenyl group was chosen for its steric and/or hydrophobic interaction with the protein residues. Moreover, the substitution on the phenyl ring of the parent molecule was selected as the hydrogen bond acceptor or hydrogen bond donor to form a bond or to impart hydrophobicity. Figure 8 and Figure 9 are complementary to each other, showing the higher lipophilic characteristics of both active sites and the most promising synthesized candidates that may be responsible for their interaction with the amino acid residues of the enzyme active pocket, especially with the presence of lipophilic moieties, such as the methoxy and chloro groups.

## 3. Materials and Methods

### 3.1. General

Melting points were measured on a Griffin melting point apparatus (Griffin, Valdosta, GA, USA) and are uncorrected. The infrared spectra were recorded as KBr discs on a Nicolet IR 200 (Thermo Fisher Scientific, Barrington, RI, USA). The ^1^H-NMR and ^13^C-NMR spectra were run using TMS as an internal standard (Aldrich chemical Co., Milwaukee, WI, USA) on a Varian Mercury VXr-300 NMR (Varian, Palo Alto, CA, USA). Mass spectra were measured on a JEOL-SX-102 instrument using electron impact ionization. Elemental analysis were performed on a Perkin-Elmer 240C analyzer (Perkin-Elmer, Norwalk, CT, USA) at the Analytical Center, College of Science, Cairo University, Egypt. All values were within ±0.4% of the theoretical values. The reactions were monitored by thin-layer chromatography (TLC) using TLC sheets precoated with UV fluorescent silica gel Merck 60 F254 plates and were visualized using a UV lamp and different solvents as mobile phases. All chemicals were purchased from (Sigma-Aldrich, Milwaukee, WI, USA).

#### 3.1.1. General Method

Title compounds **7c**–**l**, as well as intermediates, were prepared in the following steps: synthesis of 3-amino-5-(substituted phenyl)-2*H*-1,2,4-triazol (**5a**,**b**); Compound **5a**,**b** was prepared according to the literature procedure [20,21].

#### 3.1.2. General Method for Synthesis of Schiff Bases (**6c**–**l**)

A mixture of an equimolar amount of amine (**5a,b**) (0.01 mole) and the aromatic aldehyde (0.01 moles) in 50 mL acetic acid was refluxed for about 10–12 h on an oil bath. The reaction mixture was cooled, poured into ice water, extracted with ethyl acetate and, finally, dried over anhydrous sodium sulfate. The solvent was evaporated to give the solid product that was crystallized from ethyl acetate-hexane using decolorizing charcoal. The obtained compounds (**6c**–**l**) were pure enough to proceed to the next steps without further purification.

#### 3.1.3. General Method for the Synthesis of the Compounds (**7c**–**l**)

A mixture of Schiff bases (**6c**–**l**) (0.01 mol) in THF (30 mL) and mercapto acetic acid (thioglycolic acid) (0.01 mol) with a pinch of anhydrous ZnCl_2_ was refluxed for 12 h. The solvent was then removed to get a residue, which was dissolved in benzene and passed through a column of silica gel using benzene:chloroform (8:2 *v*/*v*) mixture as the eluent. The eluate was concentrated and the product crystallized using alcohol to give 4-thiazolidinones (**7c**–**l**).

*2-(4-Chlorophenyl)-3-(5-(3-chlorophenyl)-2H-1,2,4-triazol-3-yl)thiazolidin-4-one* (**7c**): Yield, 68%; m.p.: 164–166 °C. IR (KBr, cm^−1^): 3065 (CH aromatic), 1720 (C=O), 1259 (C-S). ^1^H-NMR (DMSO-*d*_6_, 300 MHz): δ = 3.22 (d,1H,-C**Hb** of CH_2_ of thiazolidinone), 4.12 (dd, *J* = 1.4 Hz, 1H, -C**ha** of CH_2_ of thiazolidinone), 5.81 (d. 1H, C**H** of thiazolidinone), 7.01–7.52 (m, 4H, Ar-H), 7.83 (dd, 4H, *J* = 7.9 Hz, 1.5, Ar-H), 8.6 (s, 1H, N**H**), ^13^C-NMR (DMSO-*d*_6_, 300 MHz): δ = 34.22 (CH_2_-C_3_H_3_NOS), 68.23 (CH-C_3_H_3_NOS), 121.77 (CH-C_6_H_5_), 124.26 (CH-C_6_H_5_), 126.83 (CH-C_6_H_5_), 127.41 (CH-C_6_H_5_), 128.19 (CH-C_6_H_5_), 128.96 (CH-C_6_H_5_), 129.18 (CH-C_6_H_5_), 129.02 (CH-C_6_H_5_), 130.17 (C-C_6_H_5_), 131.81 (C-C_6_H_5_), 132.43 (C,4Cl-C_6_H_5_), 138.76 (C,3Cl-C_6_H_5_), 163.04 (C-C_2_HN_3_), 164.71 (C-C_2_HN_3_), 175.78 (CO-C_3_H_3_NOS), MS (*m*/*z*): 391.01 [M]^+^. Analysis for C_17_H_12_Cl_2_N_4_OS (Molecular weight (m. w.) 390.01); Calcd.: C, 52.18; H, 3.09; N, 14.32. Found: C, 52.44; H, 3.13; N, 14.51.

*3-(5-(3-Chlorophenyl)-2H-1,2,4-triazol-3-yl)-2-(4-fluorophenyl)thiazolidin-4-one* (**7d**): Yield, 65%; m.p.: 170–172 °C. IR (KBr, cm^−1^): 3068 (CH aromatic), 1730 (C=O), 1256 (C-S). ^1^H-NMR (DMSO-*d*_6_, 300 MHz): δ = 4.21 (d,1H,-C**Hb** of CH_2_ of thiazolidinone), 4.34 (dd, *J* = 1.2 Hz, 1H, -C**Ha** of CH_2_ of thiazolidinone), 5.68 (d. 1H, C**H** of thiazolidinone), 7.12–7.71 (m, 4H, Ar-H), 7.91 (dd, 4H, *J* = 7.7, 1.4 Hz, Ar-H), 88.3 (s, 1H, N**H**), ^13^C-NMR (DMSO-*d*_6_, 300 MHz): δ =33.24 (CH_2_-C_3_H_3_NOS), 68.15 (CH-C_3_H_3_NOS), 121.73 (CH-C_6_H_5_), 125.11 (CH-C_6_H_5_), 126.24 (CH-C_6_H_5_), 127.64 (CH-C_6_H_5_), 128.16 (CH-C_6_H_5_), 128.79 (CH-C_6_H_5_), 129.12 (CH-C_6_H_5_), 129.75 (CH-C_6_H_5_), 130.11 (C-C_6_H_5_), 131.21 (C-C_6_H_5_), 132.03 (C,3-Cl-C_6_H_5_), 155.76 (C-C_2_HN_3_), 163.12 (C-C_2_HN_3_), 164.04 (C,4F-C_6_H_5_), 176.71 (CO-C_3_H_3_NOS), MS (*m*/*z*): 375.04 [M]^+^. Analysis for C_17_H_12_ClFN_4_OS (m. w. 374.04); Calcd.: C, 54.47; H, 3.23; N, 14.95. Found: C, 54.51; H, 3.42; N, 14.82.

*3-(5-(3-Chlorophenyl)-2H-1,2,4-triazol-3-yl)-2-(4-nitrophenyl)thiazolidin-4-one* (**7e**): Yield, 66%; m.p.: 178–180 °C. IR (KBr, cm^−1^): 3085 (CH aromatic), 1735 (C=O), 1257 (C-S). ^1^H-NMR (DMSO-*d*_6_, 300 MHz): δ = 4.09 (d,1H,-C**Hb** of CH_2_ of thiazolidinone), 4.17 (dd, *J* = 1.3 Hz, 1H, -C**Ha** of CH_2_ of thiazolidinone), 5.74 (d. 1H, C**H** of thiazolidinone), 7.11–7.62 (m, 4H, Ar-H), 7.87 (dd, *J* = 8.1, 1.7 Hz, 4H, Ar-H), 8.1 (s, 1H, N**H**), ^13^C-NMR (DMSO-*d*_6_, 300 MHz): δ =34.51(CH_2_-C_3_H_3_NOS), 68.32 (CH-C_3_H_3_NOS), 121.41 (CH-C_6_H_5_), 124.52 (CH-C_6_H_5_), 125.94 (CH-C_6_H_5_), 127.41 (CH-C_6_H_5_), 128.34 (CH-C_6_H_5_), 128.81 (CH-C_6_H_5_), 129.21 (CH-C_6_H_5_), 129.42 (CH-C_6_H_5_), 130.37 (C-C_6_H_5_), 130.13 (C,3-Cl-C_6_H_5_), 141.31 (C-C_6_H_5_), 147.28 (C,4-NO_2_-C_6_H_5_), 164.47 (C-C_2_HN_3_), 165.68 (C-C_2_HN_3_), 173.82 (CO-C_3_H_3_NOS), MS (*m*/*z*): 402.03 [M]^+^. Analysis for C_17_H_12_ClN_5_O_3_S (m. w. 401.03); Calcd.: C, 50.81; H, 3.01; N, 17.43. Found: C, 50.62; H, 3.42; N, 17.24.

*3-(5-(3-Chlorophenyl)-2H-1,2,4-triazol-3-yl)-2-(4-methoxyphenyl)thiazolidin-4-one* (**7f**): Yield, 62%; m.p.: 207–209 °C. IR (KBr, cm^−1^): 3068 (CH aromatic), 1732 (C=O), 1255 (C-S). ^1^H-NMR (DMSO-*d*_6_, 300 MHz): δ = 3.74 (s, 3H, OC**H_3_**), 4.24 (d,1H,-C**Hb** of CH_2_ of thiazolidinone), 4.38 (dd, *J* = 1.3 Hz, 1H, -C**Ha** of CH_2_ of thiazolidinone), 5.84 (d. 1H, C**H** of thiazolidinone), 7.13–7.62 (m, 4H, Ar-H), 7.92 (dd, *J* = 8.2, 1.4 Hz, 4H, Ar-H), 8.54 (s, 1H, N**H**), ^13^C-NMR (DMSO-*d*_6_, 300 MHz): δ = 34.2 (CH_2_-C_3_H_3_NOS), 54.23 (C-OCH_3_), 68.24 (CH-C_3_H_3_NOS), 116.78 (CH-C_6_H_5_), 116.82(CH-C_6_H_5_), 126.41 (CH-C_6_H_5_), 127.41 (CH-C_6_H_5_), 128.61 (CH-C_6_H_5_), 128.96 (CH-C_6_H_5_), 129.21 (CH-C_6_H_5_), 129.47 (CH-C_6_H_5_), 131.31 (C-C_6_H_5_), 133.74 (C-C_6_H_5_), 136.25 (C,3-Cl-C_6_H_5_), 157.02 (C,4-OCH_3_-C_6_H_5_), 164.22 (C-C_2_HN_3_), 164.51 (C-C_2_HN_3_), 172.89 (CO-C_3_H_3_NOS), MS (*m*/*z*): 387.06 [M]^+^. Analysis for C_18_H_15_ClN_4_O_2_S (m. w. 386.06); Calcd.: C, 55.88; H, 3.91; N, 14.48. Found: C, 55.93; H, 3.78; N, 14.27.

*3-(5-(3-Chlorophenyl)-2H-1,2,4-triazol-3-yl)-2-p-tolylthiazolidin-4-one* (**7g**): Yield, 64%; m.p.: 225–227 °C. IR (KBr, cm^−1^): 3070 (CH aromatic), 1745 (C=O), 1258 (C-S). ^1^H-NMR (DMSO-*d*_6_, 300 MHz): δ = 2.36 (s, 3H, C**H_3_**), 4.16 (d,1H,-C**Hb** of CH_2_ of thiazolidinone), 4.28 (dd, *J* = 1.2 Hz, 1H, -C**ha** of CH_2_ of thiazolidinone), 5.57 (d. 1H, C**H** of thiazolidinone) 7.08–7.79 (m, 4H, Ar-H), 7.95 (dd, 4H, *J* = 8.5, 1.8 Hz, Ar-H), 8.14 (s, 1H, N**H**), ^13^C-NMR (DMSO-*d*_6_, 300 MHz): δ = 22.78 (C-CH_3_), 35.32 (CH_2_-C_3_H_3_NOS), 60.92 (CH-C_3_H_3_NOS), 123.77 (CH-C_6_H_5_), 124.41 (CH-C_6_H_5_), 126.42 (CH-C_6_H_5_), 126.78 (CH-C_6_H_5_), 127.1 (CH-C_6_H_5_), 127.44 (CH-C_6_H_5_), 130.29 (CH-C_6_H_5_), 130.71 (CH-C_6_H_5_), 131.05 (C-C_6_H_5_), 132.47 (C,3-Cl-C_6_H_5_), 135.72 (C-C_6_H_5_), 136.47 (C-C_6_H_5_), 157.71 (C-C_2_HN_3_), 158.94 (C-C_2_HN_3_), 175.48 (CO-C_3_H_3_NOS), MS (*m*/*z*): 371.07 [M]^+^. Analysis for C_18_H_15_ClN_4_OS (m. w. 370.07); Calcd.: C, 58.30; H, 4.08; N, 15.11. Found: C, 58.45; H, 4.19; N, 14.96.

*2-(4-Chlorophenyl)-3-(5-(4-methoxyphenyl)-2H-1,2,4-triazol-3-yl)thiazolidin-4-one* (**7h**): Yield, 65%; m.p.: 211–213 °C. IR (KBr, cm^−1^): 3079 (CH aromatic), 1740 (C=O) 1256 (C-S). ^1^H-NMR (DMSO-*d*_6_, 300 MHz): δ = 3.81 (s, 3H, OC**H_3_**), 4.21 (d,1H,-C**Hb** of CH_2_ of thiazolidinone), 4.36 (dd, *J* = 1.2 Hz, 1H, -C**Ha** of CH_2_ of thiazolidinone), 5.71 (d. 1H, C**H** of thiazolidinone), 7.11–7.64 (m, 4H, Ar-H), 7.87 (dd, 4H, *J* = 8.7, 2.1 Hz, Ar-H), 8.4 (s, 1H, N**H**), ^13^C-NMR (DMSO-*d*_6_, 300 MHz): δ = 36.7 (CH_2_-C_3_H_3_NOS), 52.43 (C-OCH_3_), 69.42 (CH-C_3_H_3_NOS), 121.57 (CH-C_6_H_5_), 123,89 (CH-C_6_H_5_), 127.98 (CH-C_6_H_5_), 128.71 (CH-C_6_H_5_), 128.89 (CH-C_6_H_5_), 129.24 (CH-C_6_H_5_), 129.41 (CH-C_6_H_5_), 130.87 (CH-C_6_H_5_), 131.61 (C-C_6_H_5_), 132.54 (C,4-Cl-C_6_H_5_), 137.97 (C-C_6_H_5_), 155.4 (C,4-OCH_3_-C_6_H_5_), 156.62 (C-C_2_HN_3_), 156.81 (C-C_2_HN_3_), 174.5 (CO-C_3_H_3_NOS), MS (*m*/*z*): 387.06 [M]^+^. Analysis for C_18_H_15_ClN_4_O_2_S (m. w. 386.06); Calcd.: C, 55.88; H, 3.91; N, 14.48. Found: C, 56.04; H, 3.73; N, 14.51.

*2-(4-Fluorophenyl)-3-(5-(4-methoxyphenyl)-2H-1,2,4-triazol-3-yl)thiazolidin-4-one* (**7i**): Yield, 68%; m.p.: 215–217 °C. IR (KBr, cm^−1^): 3078 (CH aromatic), 1725 (C=O), 1259 (C-S). ^1^H-NMR (DMSO-*d*_6_, 300 MHz): δ = 3.81 (s, 3H, OCH_3_), 4.09 (d,1H,-CHb of CH_2_ of thiazolidinone), 4.18 (dd, *J* = 1.5 Hz, 1H, -CHa of CH_2_ of thiazolidinone), 5.61 (d. 1H, CH of thiazolidinone), 7.21–7.73 (m, 4H, Ar-H), 7.98 (dd, 4H, *J* = 8.5, 1.9, Ar-H), 8.2 (s, 1H, N**H**), ^13^C-NMR (DMSO-*d*_6_, 300 MHz): δ = 32.92 (CH_2_-C_3_H_3_NOS), 54.13 (C-OCH_3_), 67.83 (CH-C_3_H_3_NOS), 121.27 (CH-C_6_H_5_), 124.52 (CH-C_6_H_5_), 126.48 (CH-C_6_H_5_), 126.61 (CH-C_6_H_5_), 128.51 (CH-C_6_H_5_), 128.89 (CH-C_6_H_5_), 129.61 (CH-C_6_H_5_), 129.42 (CH-C_6_H_5_), 130.27 (C-C_6_H_5_), 132.84 (C-C_6_H_5_), 154.62 (C-C_2_HN_3_), 155.62 (C-C_2_HN_3_), 159.84 (C,4-F-C_6_H_5_), 163.81 (C,4-OCH_3_-C_6_H_5_), 174.18 (CO-C_3_H_3_NOS), MS (*m*/*z*): 371.09 [M]^+^. Analysis for C_18_H_15_FN_4_OS (m. w. 370.09); Calcd.: C, 58.37; H, 4.08; N, 15.13. Found: C, 58.46; H, 3.87; N, 15.47.

*3-(5-(4-Methoxyphenyl)-2H-1,2,4-triazol-3-yl)-2-(4-nitrophenyl)thiazolidin-4-one* (**7j**): Yield, 68%; m.p.: 218–220 °C. IR (KBr, cm^−1^): 3078 (CH aromatic), 1740 (C=O), 1257 (C-S). ^1^H-NMR (DMSO-*d*_6_, 300 MHz): δ = 3.87 (s, 3H, OC**H_3_**), 4.15 (d,1H,-C**Hb** of CH_2_ of thiazolidinone), 4.08 (dd, *J* = 1.5 Hz, 1H, -C**Ha** of CH_2_ of thiazolidinone), 5.76 (d. 1H, C**H** of thiazolidinone), 7.08–7.62 (m, 4H, Ar-H), 8.01 (dd, 4H, *J* = 8.5, 1.6 Hz, Ar-H), 8.2 (s, 1H, N**H**), ^13^C-NMR (DMSO-*d*_6_, 300 MHz): δ = 30.92 (CH_2_- C_3_H_3_NOS), 55.32 (C-OCH_3_), 65.93 (CH-C_3_H_3_NOS), 122.47 (CH-C_6_H_5_), 124.72 (CH-C_6_H_5_), 126.18 (CH-C_6_H_5_), 126.55 (CH-C_6_H_5_), 128.81 (CH-C_6_H_5_), 128.92 (CH-C_6_H_5_), 129.12 (CH-C_6_H_5_), 129.52 (CH-C_6_H_5_), 129.94 (C-C_6_H_5_), 129.98 (C-C_6_H_5_), 140.47 (C,4-NO_2_-C_6_H_5_), 153.52 (C-C_2_HN_3_), 154.87 (C-C_2_HN_3_), 164.61 (C,4-OCH_3_-C_6_H_5_), 176.28 (CO-C_3_H_3_NOS), MS (*m*/*z*): 398.08 [M]^+^. Analysis for C_18_H_15_N_5_O_4_S (m. w. 397.08); Calcd.: C, 54.40; H, 3.80; N, 17.62. Found: C, 54.64; H, 3.93; N, 17.41.

*2-(4-Methoxyphenyl)-3-(5-(4-methoxyphenyl)-2H-1,2,4-triazol-3-yl)thiazolid-din-4-one* (**7k**): Yield, 68%; m.p.: 219–221 °C. IR (KBr, cm^−1^): 3085 (CH aromatic), 1730 (C=O), 1253 (C-S). ^1^H-NMR (DMSO-*d*_6_, 300 MHz): δ = 3.75 (s, 3H, OC**H_3_**), 3.97 (s, 3H, OC**H_3_**), 4.38 (d,1H,-C**Hb** of CH_2_ of thiazolidinone), 4.23 (dd,1H, -C**Ha** of CH_2_ of thiazolidinone), 5.79 (d. 1H, C**H** of thiazolidinone), 7.21–7.72 (m, 4H, Ar-H), 8.09 (dd, 4H, *J* = 8.5, 1.6 Hz, Ar-H), 8.7 (s, 1H, N**H**), ^13^C-NMR (DMSO-*d*_6_, 300 MHz): δ = 32.26 (CH_2_-C_3_H_3_NOS), 52.44 (C-OCH_3_), 52.86 (C-OCH_3_), 67.28 (CH-C_3_H_3_NOS), 121.64 (CH-C_6_H_5_), 124.32 (CH-C_6_H_5_), 126.38 (CH-C_6_H_5_), 127.51 (CH-C_6_H_5_), 127.81 (CH-C_6_H_5_), 128.21 (CH-C_6_H_5_), 129.41 (CH-C_6_H_5_), 129.92 (CH-C_6_H_5_), 130.17 (C-C_6_H_5_), 131.56 (C-C_6_H_5_), 157.62 (C-C_2_HN_3_), 158.14 (C-C_2_HN_3_), 163.41 (C,4-OCH_3_-C_6_H_5_), 164.77 (C,4-OCH_3_-C_6_H_5_), 174.78 (CO-C_3_H_3_NOS), MS (*m*/*z*): 391.01 [M]^+^. Analysis for C_19_H_18_N_4_O_3_S (m. w. 382.11); Calcd.: C, 59.67; H, 4.74; N, 14.65. Found: C, 59.47; H, 4.93; N, 14.52.

*3-(5-(4-Methoxyphenyl)-2H-1,2,4-triazol-3-yl)-2-p-tolylthiazolidin-4-one* (**7l**): Yield, 68%; m.p.: 216–218 °C. IR (KBr, cm^−1^): 3070 (CH aromatic), 1728 (C=O), 1254 (C-S). ^1^H-NMR (DMSO-*d*_6_, 300 MHz): δ = 2.24 (s, 3H, C**H_3_**), 3.76 (s, 3H, OC**H_3_**), 4.28 (d,1H,-C**Hb** of CH_2_ of thiazolidinone), 4.21 (dd,1H, -C**ha** of CH_2_ of thiazolidinone), 5.91 (d. 1H, C**H** of thiazolidinone), 7.21–7.72 (m, 4H, Ar-H), 8.03 (dd, 4H, *J* = 8.3, 1.5 Hz, Ar-H), 8.4 (s, 1H, N**H**), ^13^C-NMR (DMSO-*d*_6_, 300 MHz): δ = 26.14 (C-CH_3_), 31.92 (CH_2_-C_3_H_3_NOS), 57.21 C-OCH_3_), 66.42 (CH-C_3_H_3_NOS), 121.57 (CH-C_6_H_5_), 124,82 (CH-C_6_H_5_), 125.88 (CH-C_6_H_5_), 126.91 (CH-C_6_H_5_), 128.11 (CH-C_6_H_5_), 128.79 (CH-C_6_H_5_), 129.61 (CH-C_6_H_5_), 129.82 (CH-C_6_H_5_), 130.07 (C-C_6_H_5_), 130.81 (C-C_6_H_5_), 134.54 (C-C_6_H_5_), 154.47 (C-C_2_HN_3_), 155.44 (C-C_2_HN_3_), 165.31 (C,4-OCH_3_-C_6_H_5_), 170.8 (CO-C_3_H_3_NOS), MS (*m*/*z*): 367.12 [M]^+^. Analysis for C_19_H_18_N_4_O_2_S (m. w. 366.12); Calcd.: C, 62.28; H, 4.95; N, 15.29. Found: C, 62.47; H, 5.14; N, 14.91.

### 3.2. Biological Evaluation

The initial antimicrobial screening (growth inhibition zone) and determination of minimum inhibitory concentration (MIC) of the tested compounds was performed by cup plate and broth dilution methods, respectively, with different strains [25,26]. Ten synthesized compounds, **7c**–**l**, were screened for their antibacterial activity against *Escherichia coli* ATCC 25922, *Proteus vulgaris* atcc 6380, *Pseudomonas aeruginosa* ATCC 27857, *Staphylococcus aureus* ATCC 29213, *Enterococcus faecalis* ATCC 29212, *Bacillus subtilis* ATCC 10400, antimycobacterial activity against *Mycobacterium fortuitum* ATCC 6841 and antifungal activity against *Candida albicans* ATCC 2091. The tested compounds were dissolved in dimethyl sulfoxide (DMSO) to obtain a stock solution (5120 μg/mL).

#### 3.2.1. Inhibition Zone Measurement Using the Cup Plate Method

Three to five pure colonies of each tested organism were taken from the overnight culture of tryptone soy agar and suspended in 5 mL of sterile normal saline. The bacterial culture was measured using the spectrophotometer (LKB^®^ Ultrospec) at 625 nm to give absorbance 0.1–0.14 (1 × 10^8^ CFU/mL). The suspension was diluted 1:100 in Cation Adjustment Mueller–Hinton (CAMH) broth (Merck^®^, Darmstadt, Germany) to obtain 1 × 10^6^ CFU/mL. The suspension was swabbed on a CAMH agar plate (Merck^®^, Darmstadt, Germany) and allowed to dry thoroughly. Then, four cups per agar plate were made using a cork borer. A 100 µL (512 μg) stock solution (5120 μg/mL) was transferred into the cup using a sterile pipette. The plates were kept in the refrigerator at 4°C for 30 min for diffusion. Then, the plates were incubated at 37 °C for 24 h, except for *Mycobacterium fortuitum* ATCC 6841, which was incubated for 48 h. After the incubation period, the diameter of the inhibition zone (including the cup diameter) was measured and recorded in mm. Ciprofloxacin, moxifloxacin and fluconazole disks BD BBL™ Sensi-Disc™ (Beckton Dickinson BBL, Franklin Lakes, NJ, USA) (BBL) were used as positive controls for antibacterial and antifungal activities, respectively. The assay was carried out in duplicate.

#### 3.2.2. Determination of MIC

MIC values were determined for the active in the cup plate method. Briefly, 1 mL of sterilized CAMH broth was dispensed into a sterile 7-mL bijou tube (Sterilin Limited, Birmingham, UK). For each compound, 14 tubes were used. Tube Numbers 13 and 14 were used as the positive growth control (no tested compound) and the negative control for the medium sterility (no microorganism), respectively. A 1 mL stock solution (5120 μg/mL) was 10-fold diluted in 9 mL CAMH to obtain 512 μg/mL. One milliliter of the tested compounds was transferred into Tube #1 and mixed well to give a concentration of 256 μg/mL. Then, 1 mL was pipetted from Tube #1 and transferred to Tube #2 to make a two-fold dilution (128 μg/mL). This procedure was repeated down to Tube #12 to reach the concentration of 0.125 μg/mL. One milliliter was discarded from Tube #12. A volume of 1 mL of inocula (1 × 10^6^ CFU/mL) was added to all tubes, except Tube #14, to give final inocula of 5 × 10^5^ CFU/mL, and the concentration of the tested compounds was two-fold diluted. Ciprofloxacin, moxifloxacin and fluconazole were used as positive controls as the antibacterial and antifungal, respectively. The inoculated tubes were incubated at 37 °C for 20 h, except for *Mycobacterium fortuitum* ATCC 6841, which was incubated for 48 h. After the incubation period, the results of the MIC were recorded manually according to the guidelines of European Committee on Antimicrobial Susceptibility testing (EUCAST), 2003.

### 3.3. Molecular Modeling Studies

The investigated compounds were subjected to surface mapping experiments using “Molecular Operating Environment” software (MOE of Chemical Computing Group Inc., on a Core i7 workstation). The molecules were built using the Builder module of MOE. Their geometry was optimized by using the Merck Molecular Force Field (MMFF94x) force field. The molecular mechanics (MM) ‏calculations *in vacuo*, dipole bond option for electrostatics, Polak–Ribiere algorithm and root mean square deviation (RMSD) gradient of 0.01 kcal/mol conformational searching in torsional space were performed using the multi-conformer method. Energy minima for the above compounds were determined by a semi-empirical method Austin Model 1 (AM1) (as implemented in MOE, 2009.10). The lowest energy conformer of each new analogue “global-minima” that was docked into the MurB enzyme-binding domain was used. The crystal structure of *Staphylococcus Aureus* MurB (PDB Entry Code 1HSK) [19] was extracted from the Brookhaven Protein Database (PDB http://www.rcsb.org/pdb). During the docking process, water molecules and all of the ligands in the crystal structures were removed, except the FAD‏ co-factor for the protein PDB Code 1HSK. All of the hydrogens were added, and the enzyme structure was subjected to a refinement protocol in which the constraints on the enzyme were gradually removed and minimized until the RMSD gradient was 0.01 kcal/mol Å. The energy minimization was carried out using the molecular mechanics force field “AMBER.” For each of the 4-thiazolidinone analogues, energy minimizations (EM) were performed using 1000 steps of steepest descent, followed by conjugate gradient minimization to an RMSD energy gradient of 0.01 Kcal/mol Å. The active site of the enzyme was defined using a radius of 10.0 Å around FAD. The energy of binding was calculated as the difference between the energy of the complex and individual energies of the enzyme and ligands [27,28,29,30]. Lowest energy aligned conformation(s) were identified. The map is color coded and indicates the following: pink, hydrogen bond; blue, mild polar; green, hydrophobic region.

## 4. Conclusions

We have reported here the design and synthesis of new 1,2,4-triazole-based thiazolidin-4-one hybrids along with their spectral and antimicrobial evaluation data. Compound **7h** followed by **7j** gave the most potent activity against *Staphylococcus aureus*. Compounds **7h**–**l** showed good antifungal activity against *Candida albicans*. The *in vitro* studies demonstrated that **7h**, **7j** and **7c** showed promising inhibitory activities against *Mycobacterium fortuitum*. Despite that the MIC values of the tested compounds were not as strong as the reported antibiotics, however, they possessed a broad spectrum of activity against Gram-positive, Gram-negative, mycobacterium and fungal strains. Our compounds are not selective, so they may be useful as moderate to high-level disinfectants. This suggests that these compounds could be formulated as disinfectants or antiseptics if they are proven later to be nontoxic to skin/mucous membranes. The search for new antibacterial agents directed towards novel targets became highly imperative. Therefore, we performed a deeper modeling study on the molecular level to clarify the proposed binding mode of these compounds into the MurB active site and consequently provide straight forward information for further structural optimization. Our results suggested that docked ligands **7h**, **7j**, **7l**, **7c**, **7i** and **7k** arranged in such a conformation with a proper and stable complex with high lipophilic interaction where the *p*-chloro or *p*-methoxy phenyl ring was embedded within the hydrophobic active site residues. Our new 1,2,4-triazole-thiazolidin-4-ones hybrids presented in this work could provide a potential for further optimization towards more active derivatives against *Staphylococcus aureus*, as well as *Candida albicans*. This study has also paved the way for generating more useful thiazolidinone analogues in future studies as bacterial MurB inhibitors.

## Figures and Tables

**Table 1:** *In vitro* antimicrobial activity of the test compounds using the inhibition zone (IZ) and MIC.

Compound No.	cLogP ^a^	Gram-negative Bacteria Gram-Positive Bacteria														Fungi	
		*Escherichia coli* ATCC 25922		*Proteus vulgaris* atcc 6380		*Pseudomonas aeruginosa* ATCC 27857		*Staphylococcus aureus* ATCC 29213		*Enterococcus faecalis* ATCC 29212		*Bacillus subtilis* ATCC 10400		*Mycobacterium fortuitum* ATCC 6841		*Candida albicans* ATCC 2091	
		IZ	MIC	IZ	MIC	IZ	MIC	IZ	MIC	IZ	MIC	IZ	MIC	IZ	MIC	IZ	MIC
**7c**	**5.12**	**17**	**64**	**Nil**	ND	**Nil**	ND	**16**	**128**	**Nil**	ND	**Nil**	ND	**20**	**64**	**Nil**	ND
**7d**	**4.55**	**Nil**	ND	**Nil**	ND	**Nil**	ND	**Nil**	ND	**Nil**	ND	**Nil**	ND	**9**	**>128**	**Nil**	ND
**7e**	**4.15**	**Nil**	ND	**Nil**	ND	**Nil**	ND	**Nil**	ND	**Nil**	ND	**Nil**	ND	**9**	**>128**	**Nil**	ND
**7f**	**4.32**	**Nil**	ND	**Nil**	ND	**Nil**	ND	**Nil**	ND	**Nil**	ND	**Nil**	ND	**10**	**>128**	**Nil**	ND
**7g**	**4.41**	**Nil**	ND	**Nil**	ND	**Nil**	ND	**Nil**	ND	**Nil**	ND	**Nil**	ND	**9**	**>128**	**Nil**	ND
**7h**	**3.84**	**30**	**16**	**Nil**	ND	**20**	**32**	**21**	**8**	**19**	**16**	**15**	**16**	**25**	**32**	**20**	**4**
**7i**	**3.44**	**19**	**64**	**Nil**	ND	**18**	**32**	**14**	**128**	**Nil**	ND	**Nil**	ND	**12**	**>128**	**19**	**16**
**7j**	**3.62**	**22**	**32**	**Nil**	ND	**16**	**32**	**16**	**16**	**Nil**	ND	**Nil**	ND	**22**	**64**	**18**	**16**
**7k**	**4.19**	**17**	**64**	**Nil**	ND	**17**	**32**	**15**	**128**	**Nil**	ND	**Nil**	ND	**Nil**	ND	**16**	**16**
**7l**	**4.91**	**23**	**32**	**Nil**	ND	**18**	**32**	**17**	**64**	**Nil**	ND	**Nil**	ND	**Nil**	ND	**20**	**16**
**CIPRO**		**27**	**≤0.25**	**25**	**≤0.25**	**29**	**≤0.25**	ND	ND	ND	ND	ND	ND	ND	ND	ND	ND
**MOX**		ND	ND	ND	ND	ND	ND	**28**	**<0.002**	**25**	**0.5**	**27**	**0.094**	**26**	**0.125**	ND	ND
**FL**		ND	ND	ND	ND	ND	ND	ND	ND	ND	ND	ND	ND	ND	ND	**28**	**0.5**

Antimicrobial activities were expressed as the inhibition zone (IZ) in millimeters (mm); Nil means (no activity) <3 mm; (weak) = 6–12 mm; (moderate) = 13–15 mm; (strong) = 16–20 mm; and (very strong) >21 mm; the experiment was carried out in triplicate, and the average zone of inhibition was calculated at a concentration of 512 μg/100 μL; MIC = minimum inhibitory concentration; MIC expressed in μg/mL; MIC was done by the cup plate method according to European Committee on Antimicrobial Susceptibility testing (EUCAST); CIPRO = ciprofloxacin; MOX = moxifloxacin, FL = fluconazole; ND = not determined, Nil = no zone of inhibition = no antimicrobial activity, ^a^ Calculated values using ChemDraw Ultra 8.0 software.
